# Pleomorphic Lobular Carcinoma in a Male Breast: A Rare Occurrence

**DOI:** 10.4061/2010/871369

**Published:** 2010-12-01

**Authors:** Bhatia Rohini, P. A. Singh, Misra Vatsala, Dhingra Vishal, Singhal Mitali, Sharma Nishant

**Affiliations:** Department of Pathology, Moti Lal Nehru Medical College, Allahabad, 211001, India

## Abstract

Carcinoma of male breast is uncommon as it accounts for 0.7% of total breast cancer. The pathology of male breast cancer is remarkably similar to that of cancers seen in women. The same histological subtypes of invasive cancer are present, although papillary carcinomas (both invasive and in situ) are more common and lobular carcinomas are less common. The predominant histological type, in males, as in females, reported in large series has been infiltrating ductal carcinoma with scattered reports of infiltrating lobular carcinoma, all of them of classical type except for a single case of pleomorphic infiltrating lobular carcinoma. Herein, we describe a case of pleomorphic lobular carcinoma occurring in male breast.

## 1. Introduction

Carcinoma arising in the male breast is a rare occurrence. The overall incidence in men is only 1% of that in women, which translates to a lifetime risk of 0.11% (as compared with about 13% in women). Risk factors are similar to those in women and include first-degree relatives with breast cancer, decreased testicular function (e.g., Klinefelter syndrome), exposure to exogenous estrogens, increasing age, infertility, obesity, prior benign breast disease, exposure to ionizing radiation, and residency in Western countries. Gynecomastia does not seem to be a risk factor. From 4% to 14% of cases in males are attributed to germline *BRCA2 *mutations. Male breast cancer accounts for 0.7% of total breast cancer [1]. Over the past 25 years, the incidence of male breast cancer has risen by 26%, from 0.86 to 1.08 per 100,000 population [2]. Approximately 85% of primary male breast carcinoma is invasive ductal carcinoma of the “no special type” subtype [3]. Carcinomas of the male breast grossly and microscopically are remarkably similar to those seen in females [4]. The predominant histological type, in males, reported in large series has been infiltrating ductal carcinoma with scattered reports of infiltrating lobular carcinoma [5], all of them were classical type except for single case of pleomorphic infiltrating lobular carcinoma [6].

## 2. Case Report

### 2.1. Clinical Details

A 55-year-old male presented with left breast mass of approximately five-month duration. There was no history of trauma, gynaecomastia, oestrogen administration, or drug use. Physical examination revealed a subareolar mass measuring 4 × 4 cm in size. Swelling was firm to hard and nontender. Overlying skin was free. There was no palpable axillary lymph node. Lumpectomy of the breast mass was done.

### 2.2. Pathological Findings

**Figure 1:**
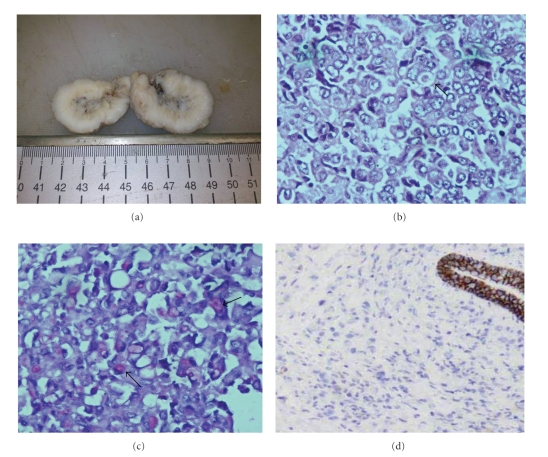
(a) Cut surface of the gross specimen shows circumscribed, grey white tumor with few small areas of necrosis. (b) Sections from the tumor mass show hyperchromatic, pleomorphic cells in diffuse sheets. At place cells with targetoid appearance (arrow) are noted [H & E x400]. (c) Higher magnification showing pleomorphic cells with PAS positive (arrow) intracytoplasmic mucin [PAS x400]. (d) Immunohistochemistry for E-cadherin negative [Avodin Biodin, x100].

On macroscopic examination, the specimen consisted of a firm grey white, circumscribed mass measuring 3 × 2.5 cm in size. Cut surface was white with a few small areas of necrosis (Figure 1(a)). 

On microscopic examination of the sections, hyperchromatic, pleomorphic cells with high nuclear cytoplasmic ratio, prominent nucleoli, and moderate amount of eosinophilic cytoplasm were seen arranged in dyscohesive sheets. Many cells showed signet-ring formation and intracytoplasmic neo-lumina with targetoid appearance (Bull's eye) (Figure 1(b)). Some cells showed with PAS positive intracytoplasmic mucin(Figure 1(c)). Immunohistochemistry for E-cadherin was done and found to be negative (Figure 1(d)). 

Radical mastectomy was done. Multiple sections processed from the mastectomy specimen were free of tumor cells. Axillary lymph nodes did not show any evidence of malignancy. Patient was well one year after the mastectomy but lost to follow up after that.

## 3. Discussion

Invasive lobular carcinoma is a distinct type of breast carcinoma based on its characteristic histological pattern. These tumors arise from the lobular and terminal duct epithelium. It occurs throughout the entire age range of breast carcinoma in adult women and usually constitutes 10% of carcinomas. Besides the classical invasive lobular other variant forms are seen. Pleomorphic lobular carcinoma, which was defined in 1992, in the female breast has a poorer prognosis than its classical counterpart [7]. This subtype demonstrates an infiltrative pattern identical to that of the classical lobular carcinoma; however, the nuclear features show greater pleomorphism, increased chromatin clumping, single or multiple prominent nucleoli, and usually abundant cytoplasm. The degree of nuclear atypia may approach that which is found in infiltrating ductal carcinoma, but the invasive pattern characteristic of the classical lobular variant is always well maintained. Weidner et al. have also reported a similar pattern of growth in this tumor as that of a classical lobular breast carcinoma, but it exhibited marked degree of nuclear pleomorphism and abundant cytoplasm [8]. Similar findings were noted in our case. It, also frequently, exhibits apocrine differentiation, which, however, was not seen in our case. Signet ring morphology is more common than the classical counterpart and similar findings were noted in our case. 

Immunohistochemical staining for E-cadherin may help in diagnosis [9, 10]; its expression is lost in infiltrating lobular carcinoma as seen in our case. Compared with classical invasive lobular carcinoma, pleomorphic lobular carcinoma shows significantly higher Ki67 index, lower estrogen receptor and progesterone receptor expression, and higher incidence of HER2 gene amplification. Both classical invasive lobular and pleomorphic lobular carcinoma demonstrate loss of 16q and gain of 1q, in the majority of the cases [11]. 

 Pleomorphic lobular carcinoma is a very aggressive tumor and is known to recur. Since grading of lobular carcinoma is difficult, recognition of the pleomorphic subtype is useful in identifying a lethal variant. Its behavior is assessed on the basis of tumor size at presentation and the frequency of nodal metastases [12].

##  Conflict of Interest

The authors declare that they have no conflict of interest.

##  Consent

The study was approved by the ethical committee.
